# Replicating and projecting the path of COVID-19 with a model-implied reproduction number

**DOI:** 10.1016/j.idm.2020.08.007

**Published:** 2020-08-28

**Authors:** Shelby R. Buckman, Reuven Glick, Kevin J. Lansing, Nicolas Petrosky-Nadeau, Lily M. Seitelman

**Affiliations:** Federal Reserve Bank of San Francisco, 101 Market Street, San Francisco CA, 94105, USA

**Keywords:** Coronavirus, COVID-19, SEIR Model, Epidemics, Reproduction number

## Abstract

We demonstrate a methodology for replicating and projecting the path of COVID-19 using a simple epidemiology model. We fit the model to daily data on the number of infected cases in China, Italy, the United States, and Brazil. These four countries can be viewed as representing different stages, from later to earlier, of a COVID-19 epidemic cycle. We solve for a model-implied effective reproduction number $R_{t}$ each day so that the model closely replicates the daily number of currently infected cases in each country. For out-of-sample projections, we fit a behavioral function to the in-sample data that allows for the endogenous response of $R_{t}$ to movements in the lagged number of infected cases. We show that declines in measures of population mobility tend to precede declines in the model-implied reproduction numbers for each country. This pattern suggests that mandatory and voluntary stay-at-home behavior and social distancing during the early stages of the epidemic worked to reduce the effective reproduction number and mitigate the spread of COVID-19.

## 1 Introduction

As of July 19, 2020, the ongoing COVID-19 pandemic has infected nearly 15 million people worldwide, accounting for over 600,000 deaths.^1^ The two hardest hit nations are the United States and Brazil, as measured by the total number of confirmed cases. In recent months, epidemiology models have been used to project the path of the epidemic in different locations and help guide decisions about public health interventions.^2^

This paper demonstrates a methodology for replicating and projecting the path of COVID-19 using a simple epidemiology model. We fit a standard compartmental epidemiology model (called a SEIR model) to daily data on the number of COVID-19 infected cases and closed cases (recovered or deceased) in four countries: China, Italy, the United States, and Brazil.^3^ These four countries can be viewed as representing different stages, from later to earlier, of a COVID-19 epidemic cycle. China (specifically Hubei Province) has experienced a nearly complete epidemic cycle in which the number of COVID-19 infected cases dropped to a value of only 55 on June 10.^4^ Italy is three months beyond its peak number of infected cases that occurred on April 19. The number of infected cases in both the United States and Brazil continue to increase. In the United States, the number of infected cases reached a local peak on May 30. But after trending down for five days, the number of infected cases reversed course and has continued to rise through the end of our data sample on July 19. The trailing 7-day average daily growth rate of infected cases in the United States started trending up in the first week of June, but has recently leveled off at a value near 1.5%. In Brazil, the trailing 7-day average daily growth rate of infected cases is also near 1.5%, but the growth rate is more volatile than in the United States.

In addition to representing different stages of the COVID-19 epidemic, the four countries that we examine represent different magnitudes in the total number of cases (infected plus closed). China has recorded only about 84,000 total cases, whereas Italy has nearly three times that number. In contrast, the total number of cases in the United States and Brazil are currently about 3.9 million and 2.1 million, respectively.

Based on epidemiological evidence, we calibrate the incubation period for COVID-19 (the average time between exposure and subsequent infection) to be 5.1 days for each country. Based on the nearly complete epidemic cycle for China, we calibrate the illness duration parameter (the average time between infection and either recovery or death) to be 20 days for each country. This value allows the SEIR model’s law of motion for China to approximately match the end-of-sample number of closed cases on July 19. We introduce an additional country-specific parameter in the law of motion for closed cases so that we can exactly match the end-of-sample smoothed number of closed cases in each country. The additional parameter allows us to capture cross-country differences in the reporting of recoveries or deaths that can influence the transition rate from infected cases to closed cases. For the out-of-sample projections, we assume that the additional parameter converges towards 1.0 in a manner that approximates the quasi-real time trajectory of the calibrated value for China.

Given the model parameter values, we solve for the model-implied reproduction number $R_{t}$ each day so that our SEIR model exactly replicates a centered 7-day moving average of the number of infected cases in each country. We use smoothed data in place of the raw data for this computation because it helps to reduce the sensitivity of the model’s out-of-sample projections to daily fluctuations in new infected cases. But in-sample, the model continues to closely replicate the raw number of infected and closed cases in each country.

During the early stages of the epidemic, the model-implied $R_{t}$ is typically large and volatile to capture the rapid and uneven growth in the number of infected cases. But as the epidemic progresses, the model-implied $R_{t}$ tends to decline and become less volatile, providing a daily indicator that can track the degree to which mandatory or voluntary actions by individuals may be helping to mitigate the spread of the disease. Our model-implied reproduction number should not be interpreted literally as the average number of secondary infections per infected case, as usually defined in the epidemiology literature. Rather, the model-implied reproduction number can be interpreted as the analog to the “Solow residual” in economics, acting as a stand-in for whatever time-varying model complexities are needed to closely replicate the observed time series of infected cases.^5^

For the out-of-sample projections, we fit a behavioral function to the in-sample data that allows for the endogenous response of $R_{t}$ to movements in the lagged number of infected cases. The function captures the idea that a rising number of infections will trigger a behavioral response by individuals or health authorities that helps to mitigate the spread of the disease. Our methodology allows us to make projections about the future path of the epidemic while closely replicating the in-sample data. Nevertheless, we wish to emphasize that our out-of-sample projections are subject to enormous uncertainty and can sometimes shift by large amounts from one week to the next, depending on recent incoming data. We illustrate this important point with a quasi real-time experiment in which we plot a sequence of out-of-sample projections for China and the United States using different end-of-sample starting points for the projections. Given the wide range of estimates for COVID-19 fatality rates, we do not attempt to separately project recoveries versus deaths, but we do report some statistics on closed case fatality rates and estimates of more refined fatality rates from other studies.

The COVID-19 scenarios examined here are intended to demonstrate our methodology and provide a qualitative view of potential epidemic trajectories in a small sample of selected countries. The out-of-sample projections should not be viewed as definitive forecasts.^6^ At the end of our raw data sample on July 19, the epidemic cycle in China appears nearly complete with only 251 infected cases. For Italy on July 19, there are about 12,400 infected cases and about 232,000 closed cases. The projected number of closed cases for Italy at the end of the epidemic is around 260,000.

For the United States on July 19, there are about 1.953 million infected cases and about 1.946 million closed cases. Our model projects the peak number of infections in the United States to occur on or about August 8. This projection reflects what might be called a “resurgent first wave” because a plot of the actual and projected number of infections exhibits a double-peaked shape. The projected number of closed cases for the United States at the end of the epidemic is 8.89 million. For Brazil on July 19, there are about 649,000 infected cases and about 1.45 million closed cases. Our model projects the peak number of infections in Brazil to occur on or about August 10. The projected number of closed cases for Brazil at the end of the epidemic is 4.45 million.

Finally, we show that declines in measures of population mobility tend to precede declines in the model-implied reproduction numbers for each country. This pattern suggests that mandatory and voluntary stay-at-home behavior and social distancing during the early stages of the epidemic worked to reduce the effective reproduction number and mitigate the spread of COVID-19. More recently, measures of population mobility have been trending upwards in all four countries. This pattern reflects both the relaxation of mandatory containment measures and increased voluntary mobility. As of July 19, a resurgence of new infections in some areas of the United States has triggered a reinstatement of some containment measures, consistent with our behavioral hypothesis. At the end of our data sample, measures of population mobility for the United States appear to have plateaued at a level that is below the pre-epidemic baseline.

### Related literature

The number of new COVID-19 related research papers is growing in a manner that may rival the growth rate of the disease itself. It is not possible to summarize the many related contributions to the literature, whether in epidemiology, economics, or other fields. Nevertheless, we wish to highlight some known contributions that employ methods that appear closely related to our approach.

Kucinskas (2020) and Arroyo-Marioli, Bullano, & Rondon-Moreno, 2020 employ SIR models and data on the number of infected cases to infer the time path of the effective reproduction number in various countries using a Kalman filter that treats the reproduction number as an unobserved component. Beenstock & Dai, 2020 compute daily values of the effective reproduction number in various countries using a “perpetual inventory method” that cumulates the number of infected cases over time while assuming a fixed period of contagiousness for each infected case. Dandekar & Barbastathis, 2020 allow for time variation in their SEIR model-implied reproduction number by introducing a new variable called the “strength of quarantine.” They solve for the time path of the unobserved quarantine variable and other parameters to produce a best fit of the number of infected and recovered cases in various locations. Toda (2020) estimates values of the COVID-19 transmission rate for many countries by fitting a SIR model to daily data on the fraction of confirmed cases in the population.

As discussed by Ma (2020), “phenomenological models,” or curve-fitting approaches, represent an alternative to epidemiology models when forecasting the evolution of an epidemic. An influential example of this approach applied to COVID-19 is the model developed by the University of Washington’s Institute for Health Metrics and Evaluation (Murray, 2020). Other recent examples include Roosa et al. (2020), Li and Linton (2020), Liu, Moon, and Schorfheide (2020), and Harvey & Kattuman, 2020.

A COVID-19 forecasting model developed by Atkeson et al. (2020) combines a curve-fitting approach with a simple SIRD model. Specifically, they fit a smooth curve to daily data on the cumulative number of deaths in a given location and then solve for the values of the model parameters (including initial conditions) and time paths of the model variables (including the effective reproduction number) so as to exactly replicate the smoothed curve of cumulative deaths. Fernandez -Villaverde & Jones, 2020 adopt a similar approach by inverting a simple SIRD model to solve for the time path of the effective reproduction number that causes the model to replicate the smoothed number of cumulative and daily deaths in various locations. In both papers, the numbers of infected and recovered cases are inferred from the model; only the number of deaths is considered observable. In contrast, our approach closely replicates the numbers of infected and closed cases (recovered or deceased) in the data.^7^ In reality, data on the number of infections, recoveries, or deaths are all measured with error, so in the end, it comes down to which variables the model builder chooses to replicate.

Atkeson (2020a) and Stock (2020) present epidemiology model simulations for different “flattening the curve” strategies that define the out-of-sample trajectory of the effective reproduction number. Eichenbaum et al. (2020), among a long list of others, explicitly model the welfare-maximizing choices of individuals and policymakers that, in turn, influence the economic and epidemiological consequences of the disease.

Atkeson (2020b), Korolev (2020), and Fernandez -Villaverde & Jones, 2020 each demonstrate that different sets of epidemiology model parameters can fit the in-sample data equally well, yet imply markedly different long run forecasts. Our quasi real-time projections make a similar point. Hong et al. (2020) consider an epidemiology model in which the effective reproduction number is subject to stochastic shocks. They show that, relative to the deterministic version of the same model, the stochastic version can predict a substantially lower number of infections, even at horizons beyond 12 months.

The remainder of the paper is organized as follows. Section 2 presents the model, followed by the derivation of the model-implied reproduction number in section 3. The data, parameter values, and initial conditions are discussed in section 4. Section 5 shows time series plots of the model-implied reproduction numbers for China, Italy, the United States, and Brazil. Out-of-sample projections for each country are presented in section 6. Time series plots of population mobility indices versus model-implied reproduction numbers are presented in section 7. The appendix outlines an extended version of our model that includes asymptomatic infected cases.

## 2 Model

The canonical SEIR model of epidemics divides the population *N* into 4 compartments: Susceptible $S_{t}$, Exposed $E_{t}$ (but not yet infected due to an incubation period), Infected $I_{t}$, and Removed (or Resolved) $R_{t}$, representing closed cases, i.e., those who are either recovered or deceased.^8^ Homogeneous random mixing between susceptible and infected individuals creates exposed individuals who later fall ill at the end of a disease incubation period. Infected individuals experience a period of illness, after which they may either recover or die. At the beginning of an epidemic, the share of the population susceptible to infection is high. The share of the population that is infected accelerates as each infected person can infect more than one other person. The number of new infected cases eventually slows as there are fewer susceptible individuals to infect and more individuals who have become non-infectious because they recover or die. The basic model employed here does not separate recoveries from deaths.

The propagation of an epidemic depends crucially on the daily transmission rate $\beta_{t}$. The value of $\beta_{t}$ may be influenced by public health measures known as non-pharmaceutical interventions (NPIs) or by the endogenous response of the population as awareness of the disease grows.^9^ Other model parameters include σ, the rate at which exposure leads to infection (the inverse of the incubation period) and γ, the rate of recovery or death (the inverse of the illness duration). Epidemiological models frequently refer to a “basic reproduction number,” denoted by $R_{0}$ $\equiv \beta_{0}/\gamma$. This is the number of secondary infections that one infected case produces in a fully susceptible population at t=0 through the duration of the infectious period (given by 1/γ). As the epidemic evolves $\left(t\;>\;0\right)$, the number of susceptible individuals in the population is reduced. For t>0, we define the effective reproduction number as $R_{t}\equiv \beta_{t}/\gamma$ (also called the normalized transmission rate) which measures the average number of secondary infections per infected case in a population that is no longer fully susceptible.^10^ When $R_{t}\;>\;1$, the number of infected cases continues to grow until the disease eventually spreads to nearly the entire population. However, when $R_{t}\;<\;1$, the growth rate of infected cases is slow enough so that the disease eventually dies out before reaching a large fraction of the population.

Given parameter values and a set of initial conditions $I_{0}$, $E_{0},$ $R_{0},$ and $S_{0}=N-I_{0}-E_{0}-R_{0}$, the four health compartments evolve according to the following laws of motion:

(1) $$S_{t}=S_{t-1}-R_{t}\gamma \frac{S_{t-1}I_{t-1}}{N},$$

(2) $$E_{t}=\left(1-\sigma \right)E_{t-1}+R_{t}\gamma \frac{S_{t-1}I_{t-1}}{N},$$

(3) $$I_{t}=\left(1-\gamma \right)I_{t-1}+\sigma E_{t},$$

(4) $$R_{t}=R_{t-1}+\theta_{T}\gamma I_{t-1},$$

where we have made the substitution $\beta_{t}=R_{t}\gamma$ into equations (1), (2). The ratio $S_{t-1}/N$ is the recent fraction of the population that is susceptible to the disease. This ratio will be close to 1 during the initial stages of an epidemic like COVID-19 for which the population has little or no herd immunity.^11^ To facilitate the computation of a model-implied value of $R_{t},$ we postulate that the daily number of exposed cases $E_{t}$ in equation (2) immediately impacts the daily number of infected cases $I_{t}$ in equation (3).^12^

In equation (4), we introduce the additional parameter $\theta_{T}\;>\;0.$ This parameter allows the model to capture country-specific differences in the reporting of recoveries or deaths that can influence the transition rate from infected to closed cases.^13^ In-sample, we calibrate the value of $\theta_{T}$ for each country so that the model exactly matches the end-of-sample smoothed number of closed cases, denoted by$\;R_{T}.$ For the out-of-sample projections (t>T), we assume that $\theta_{t}$ converges towards 1.0 according to the following law of motion:

(5) $$\theta_{t}=\theta_{t-1}+\kappa \theta_{t-1}\left(1-\theta_{t-1}\right),$$

where κ>0 governs the speed of convergence. We estimate the value of κ using the quasi real-time evolution of the calibrated value of *θ*_*T*_ for China, which has gone through a nearly complete COVID-19 epidemic cycle.^14^

As described below, we fit the above model to smoothed data on the number of COVID-19 infected and closed cases in China, Italy, the United States, and Brazil. We then project the out-of-sample path of the epidemic using a behavioral function that governs the evolution of $R_{t}.$

## 3 Model-implied reproduction number

Starting from equation (1), (2), (3), and then solving for $R_{t}$ yields the following model-implied value of the reproduction number:

(6) $$R_{t}=\frac{\sigma^{-1}\left[I_{t}-\left(1-\gamma \right)I_{t-1}\right]-\left(1-\sigma \right)E_{t-1}}{\gamma S_{t-1}I_{t-1}/N},$$

which is not influenced by the additional parameter $\theta_{T}.$ Given values for σ, γ, and *N*, together with the initial conditions of the model variables, we use equation (6) to solve for the value of $R_{t}$ each day for t=1,2,3... so that the model exactly replicates a centered 7-day moving average of the number of infected cases in the data for the in-sample period. Specifically, the values of $I_{t}$ and $I_{t-1}$ in equation (6) are taken from the smoothed data which runs through July 16. We use smoothed data for $I_{t}$ and $I_{t-1}$ because this helps to reduce the sensitivity of the model’s out-of-sample projections (described below) to daily fluctuations in new infected cases. But in-sample, the model continues to closely replicate the raw number of infected and closed cases in each country.

**Fig. 1:**
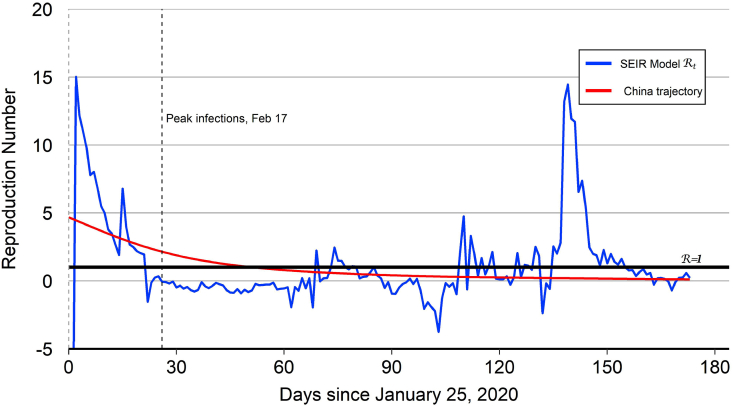
China reproduction number. *Notes*: The peak number of infections for China occurred on February 17 (t=26). After this date, the model-implied $R_{t}$ tracks mostly below 1.0 aside from some brief daily fluctuations. The spike in the model-implied $R_{t}$ around t=140 reflects an outbreak of new cases in the capital city of Beijing.

During the early stages of the epidemic when the value of the denominator in equation (6) is low (because $I_{t-1}$ is low and $S_{t-1}/N$ ≈1 ), the model-implied reproduction number is typically large (i.e., $R_{t}\gg 1$) and volatile to capture the rapid and uneven growth in the number of infected cases.^15^ As the epidemic progresses, the quantity $S_{t-1}I_{t-1}/N$ in the denominator increases and the model-implied reproduction number tends to decline and become less volatile. During the progression stage, the model-implied reproduction number can serve as a daily indicator that can track the degree to which mandated or voluntary behavior on the part of individuals in the population may be helping to mitigate the spread of the disease. Towards the end of the epidemic cycle when the quantity $S_{t-1}I_{t-1}/N$ again becomes low, the model-implied reproduction number can once again become more volatile. We can see examples of this end-of-cycle volatility in Fig. 1 for China. But in these late stages of the cycle, the model-implied $R_{t}$ has already served its purpose in tracking the daily progression of the disease.

In the appendix, we consider an extended version of the model that allows a fraction of infected cases to be asymptomatic. We show that a model that does not explicitly account for asymptomatic cases when they are indeed present can exhibit a larger model-implied reproduction number, thus capturing the impact of the asymptomatic cases in a reduced-form way.

## 4 Data, initial conditions, and parameter values

Raw data for the daily number of infected (or active) cases and closed cases (recovered or deceased) are from www.worldometers.info/coronavirus/ 16 Starting from the raw data ending on July 19, we apply a centered 7-day moving average to construct the time series for $I_{t}$ that is used to compute $R_{t}$ from equation (6). For China, we use January 25, 2020 to represent t=0. For Italy and the United States, we use February 25, 2020 to represent t=0. For Brazil, we use March 1, 2020 to represent t=0. These dates allow for some smoothing of the raw data before computing the initial model-implied reproduction numbers. Given that our raw data sample runs through July 19, the endpoint *T* of the smoothed data is July 16.

We calibrate *N* to equal the total population of each country with the exception of China, where *N* equals the population of Hubei Province, the area that accounts for nearly all confirmed cases. The values of $I_{0}$ and $R_{0}$ are the smoothed number of infected and closed cases at t=0. Following Atkeson (2020a), we set $E_{0}=4I_{0}$ in all four countries, such that $S_{0}=N-5I_{0}-R_{0}.$ Based on a recent study of COVID-19 cases in China by Lauer et al. (2020), we set σ=1/5.1 in all four countries, implying an average incubation period of 5.1 days.

When $\theta_{T}=1,$ the model’s law of motion for closed cases, equation (4), implies $\gamma =\left(R_{T}-R_{0}\right)/\Sigma_{t=0}^{T-I}I_{t}$, where $R_{T}$ is the *smoothed* number of closed cases at the end of our data sample on day *T* and the denominator is the cumulative sum of smoothed infected cases through day T−1. Using this formula, we obtain γ≈1/20 for China, which is the only country so far to have experienced a nearly complete COVID-19 epidemic cycle. Based on this result, we set γ=1/20 for all countries, implying an illness duration of about three weeks on average.

**Figure A.1:**
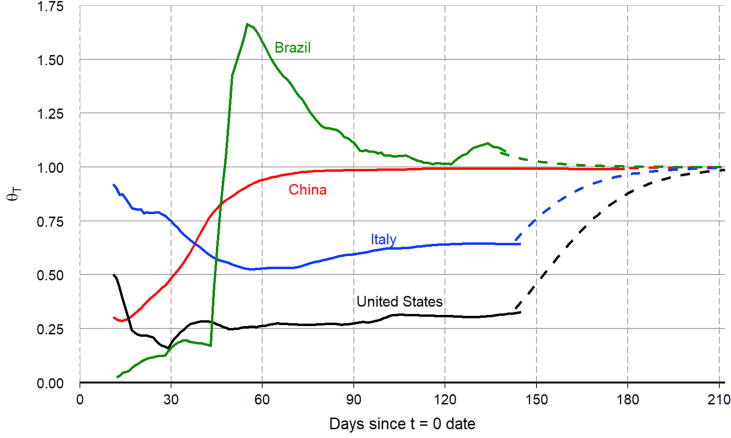
Calibrated value of parameter $\theta_{T}$. *Notes:* Given the common value of γ = 1/20 for all countries, we solve for the value of *θ*_*T*_ so that the model predicted value of *R*_*T*_ exactly matches the end-of-sample smoothed number of closed cases for each country. The figure plots the quasi-real time evolution of $\theta_{T}$ for each country. For the out-of-sample projections (*t* > *T*), we assume that $\theta_{t}$ converges towards 1.0, as governed by equation (5) with κ = 0.07, which is estimated from the quasi-real time evolution of $\theta_{T}$ for China. The dashed lines show the out-of-sample paths of $\theta_{t}$ for each country.

Given the common value of γ=1/20, we solve for the value of $\theta_{T}$ so that the model-predicted value of $R_{T}$ exactly matches the end-of-sample smoothed number of closed cases in each country. Specifically, we set $\theta_{T}=\left(R_{T}-R_{0}\right)/\Sigma_{t=0}^{T-I}\gamma I_{t}$. For China, we obtain $\theta_{T}\approx 1$ by construction. For Brazil, we obtain $\theta_{T}=1.07,$ implying a somewhat faster transition rate from infected to closed cases. But for Italy and the United States we obtain $\theta_{T}=0.64$ and $\theta_{T}=0.33,$ respectively, implying slower transition rates from infected to closed cases. These faster or slower transition rates may reflect the lack of uniform standards for the reporting of recoveries among local, state, or national governments.^17^ But death counts can also be inaccurate, as evidenced by the April 17 revision to the number of COVID-19 deaths in Wuhan, China, which caused the number to jump from 2,579 to 3,869, an increase of 50%.^18^ Figure A.1 in the appendix plots the quasi real-time evolution of $\theta_{T}$ for each country. For the out-of-sample projections, we estimate the value of the speed-of-convergence parameter κ in equation (5) using the quasi real-time evolution of $\theta_{T}$ for China. The estimation yields κ=0.07 with a standard error of 0.01.

To construct model projections for the out-of-sample paths of $I_{t}$ and $R_{t}$, we must project the future evolution of the effective reproduction number $R_{t}$. Along the lines of Eksin et al. (2019) and Cochrane (2020), we postulate a behavioral function that allows for the endogenous response of $R_{t}$ to movements in the number of infected cases. Specifically, we assume that the out-of-sample value of $R_{t}$ evolves according to the law of motion

(7) $$R_{t}=R_{t-1}I_{t-1}^{-\eta },$$

where η>0. Equation (7) implies that the out-of-sample reproduction number is highly persistent, but it responds negatively to an increase in the lagged number of infected cases. This function captures the idea that a rising number of infections will trigger a behavioral response by individuals or health authorities that helps to mitigate the spread of the disease. A number of recent COVID-19 studies present empirical evidence in support of this type of behavioral response (Goolsbee & Syverson, 2020; Hatzius et al., 2020; Maloney & Taskin, 2020; Winkler, 2020).^19^

Given the in-sample time path of the model-implied $R_{t}$, we solve for the best fit values of the starting reproduction number $R_{0}$ and the behavioral response parameter η that cause the end-of-sample value of $R_{t}$ computed from equation (7) to hit an end-of-sample target value.^20^ For Italy, the United States, and Brazil, the end-of-sample target value is the model-implied $R_{t}$ from equation (6) averaged over the most recent 7 days. As before, using a 7-day average helps to reduce the sensitivity of the out-of-sample projections to daily fluctuations in new infected cases. For China, we set the end-of-sample target value to 0.1, reflecting our view that the epidemic cycle in Hubei Province is nearly complete. Otherwise, the end-of-sample target value can be unduly influenced by the end-of-cycle volatility in the model-implied $R_{t},$ as evidenced in Fig. 1.^21^ For the first out-of-sample projection, we set $R_{t-1}$ in equation (7) equal to the end-of-sample target value for each country.

Table 1 summarizes the initial conditions and parameter values used in the projections.

**Table 1:** Initial conditions and parameter values.

Country	t=0	*N*	$I_{0}$	$E_{0}$	$R_{0}$	$\theta_{T}$	$R_{0}$	η
China (H.P.)	Jan 25	60$\times 10^{6}$	2443.4	$4I_{0}$	113.1	0.99	4.8	0.0031
Italy	Feb 25	62$\times 10^{6}$	375.3	$4I_{0}$	25.0	0.64	6.0	0.0014
United States	Feb 25	332$\times 10^{6}$	45.9	$4I_{0}$	6.0	0.33	9.7	0.0011
Brazil	Mar 1	212$\times 10^{6}$	1.9	$4I_{0}$	0.0	1.07	11.4	0.0014

*Notes*: For all countries, σ=1/5.1, γ=1/20, and κ=0.07. The values of $\theta_{T}$, $R_{0}$ and η are computed using smoothed data that runs through *T =* July 16. H.P. = Hubei Province.

## 5 Model-implied reproduction numbers for each country

Since China (specifically Hubei Province) has experienced a nearly complete COVID-19 epidemic cycle, it offers a template for modeling the evolution of the epidemic in other countries. The model-implied $R_{t}$ for China together with the “China trajectory” are plotted in Fig. 1. The level and volatility of the model-implied $R_{t}$ for China is high at beginning stages of the epidemic cycle when the quantity $S_{t-1}I_{t-1}/N$ in the denominator of equation (6) is low. But during the middle stage of the epidemic, the volatility of the model-implied $R_{t}$ is low. The peak number of infections for China occurred on February 17 (t=26). After this date, the model-implied $R_{t}$ tracks mostly below 1.0 aside from some noisy fluctuations that derive from changes in the small number of infected cases toward the end of the epidemic. The end-of-sample spike in the model-implied $R_{t}$ for China reflects a recent outbreak of new COVID-19 cases in Beijing, as noted in the introduction.

The China trajectory that is used for out-of-sample projections is the estimated version of equation (7) with $R_{0}=4.8$ and η=0.0031. While the starting value $R_{0}$ may seem rather large, a study by Aguiar et al. (2020) argues that the rapid exponential growth of recorded COVID-19 cases in thirteen countries during February 2020 and March 2020 implies a very high percentage of asymptomatic carriers. Their model implies that the effective reproduction number at the start of the outbreak could range from 5.5 to 25.4, with a point estimate of 15.4.^22^

**Fig. 2:**
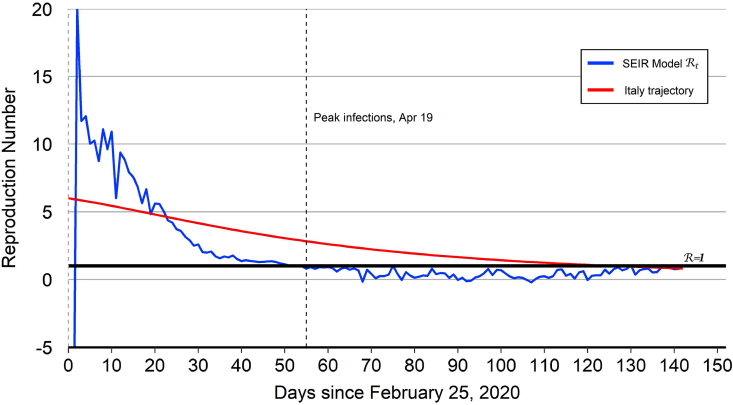
Italy reproduction number. *Notes*: The peak number of infections for Italy occurred on April 19 (t=54). After this date, the model-implied $R_{t}$ tracks below 1.0.

The model-implied $R_{t}$ for Italy together with the “Italy trajectory” are plotted in Fig. 2. As with China, the level and volatility of the model-implied $R_{t}$ are high during the first 25 days of the epidemic.^23^ The peak number of infections for Italy occurred on April 19 (t=54). Compared to China, it took longer for Italy to reach its peak number of infections. The model-implied $R_{t}$ for Italy tracks below 1.0 after the infection peak, reflecting the persistent decline in the number of infected cases. The Italy trajectory that is used for the out-of-sample projections starts at $R_{0}=6.0$ and then declines over time to hit the end-of-sample target value of 0.81.

**Fig. 3:**
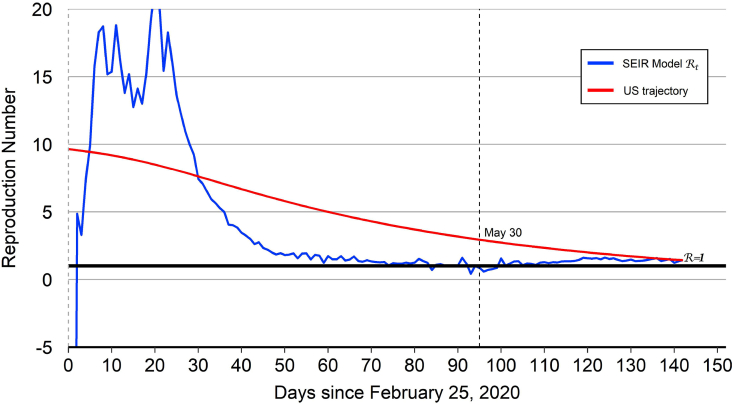
United States reproduction number. *Notes*: The model-implied $R_{t}$ for the United States dropped below 1.0 from May 30 (t=95) through June 3 (t=99), reflecting a short-lived decline in the number of infected cases. But from June 4 onward, the model-implied $R_{t}$ for the United States has remained above 1.0, reflecting an upward trend in the number of infected cases.

The model-implied $R_{t}$ for the United States together with the “United States trajectory” are plotted in Fig. 3. As with China and Italy, the level and volatility of the model-implied $R_{t}$ for the United States are high during the first 25 days of the epidemic. But the level and volatility both decline noticeably thereafter. Indeed, the model-implied $R_{t}$ dropped below 1.0 from May 30 through June 3, reflecting a short-lived decline in the number of infected cases. But from June 4 onward, the model-implied $R_{t}$ for the United States has remained above 1.0, reflecting an upward trend in the number of infected cases. The United States trajectory that is used for out-of-sample projections starts at $R_{0}=9.7$ and then declines over time to hit the end-of-sample target value of 1.42. The United States trajectory crosses below 1.0 on August 7 (t=164), one day before the projected date of peak infections on August 8.

**Fig. 4:**
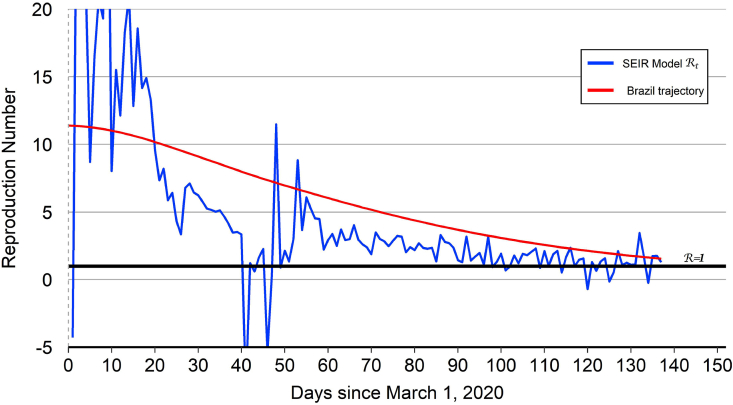
Brazil reproduction number. *Notes*: The model-implied $R_{t}$ for Brazil exhibits some sharp downward and upward jumps during the middle part of April (t=40 to t=50), which may reflect reporting errors in the number of infected cases. The model-implied $R_{t}$ averaged over the most-recent 7 days remains above 1.0 at the end of our data sample, reflecting an upward trend in the number of infected cases.

The model-implied $R_{t}$ for Brazil together with the “Brazil trajectory” are plotted in Fig. 4. As with the other countries, the level and volatility of the model-implied $R_{t}$ are high during the first 25 days of the epidemic. But after an interval where the level and volatility are both declining, the model-implied $R_{t}$ for Brazil exhibits some sharp downward and upward jumps during the middle part of April (t=40 to 50), which reflect corresponding jumps in the number of infected cases in the data. These jumps may reflect reporting errors or corrections to reporting errors.^24^ Since then, however, the level and volatility of the model-implied $R_{t}$ have resumed their declines. The Brazil trajectory that is used for out-of-sample projections starts at $R_{0}=11.4$ and then declines over time to hit the end-of-sample target value of 1.56. The Brazil trajectory crosses below 1.0 on August 9 (t=161), one day before the projected date of peak infections on August 10. Based on this trajectory, Brazil appears roughly aligned with the United States in the COVID-19 epidemic cycle. During the month of May, it had appeared that Brazil was about two to three weeks *behind* the United States in the cycle. But the incoming data during the months of June and July has served to delay the projected date of peak infections for the United States.

## 6 Out-of-sample projections

Using the foregoing framework, we construct out-of-sample projections for the number of infected cases and the number of closed cases (recovered or deceased) in each country. In-sample, we assume that $R_{t}$ is given by the country’s model-implied value that is computed using smoothed data that runs through July 16. For the out-of-sample projections starting on July 17, we assume that $R_{t}$ evolves according to the estimated version of equation (7).

### 6.1 China

**Fig. 5:**
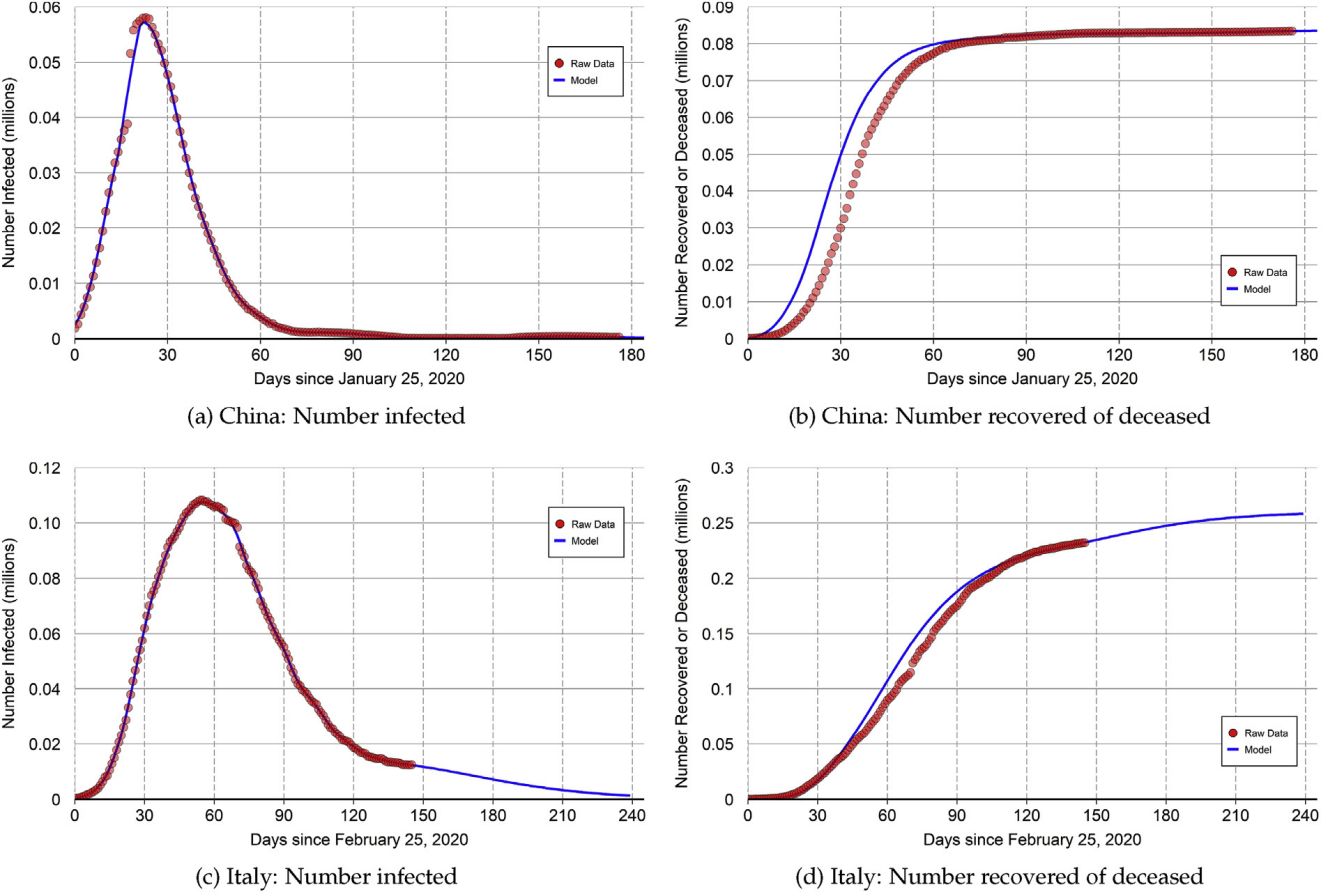
Out-of-sample projections: China and Italy. *Notes*: The top panels show the out-of-sample projections for China (specifically Hubei Province). The peak number of infections occurred on February 17 (t=26). At the end of our data sample, the epidemic cycle is nearly complete with only a small number of infected cases. The bottom panels show the out-of-sample projections for Italy. The peak number of infections occurred on April 19 (t=54). The projected number of closed cases for Italy at the end of the epidemic is around 260,000.

The top panels of Fig. 5 show the out-of-sample predictions for China. At the end of our data sample, the epidemic cycle in Hubei Province appears nearly complete with only a small number of infected cases. The most-recent recorded death from COVID-19 occurred on May 17. The peak number of infections occurred on February 17 (t=26) at 58,016. By construction, the model closely replicates the number infected cases (top left panel) and the number of closed cases (top right panel).

Even though COVID-19 emerged just a few weeks prior to the Chinese New Year (a period of typically high travel), the rapid deployment of NPIs proved to be effective in limiting the spread of the outbreak. This is a remarkable achievement for an area with a population of around 60 million people.^25^ A study by Lai et al. (2020) concludes that “if NPIs were conducted one week, two weeks, or three weeks later, the number of cases could have shown a 3-fold, 7-fold, and 18-fold increase across China, respectively.”^26^ The same study acknowledges that “If NPIs could have been conducted one week, two weeks, or three weeks earlier in China, [then] cases could have been reduced by 66%, 86%, and 95%, respectively.”

At the end of our data sample, China has recorded a total of 4634 deaths out of 83,660 closed cases, yielding a closed case fatality rate of 5.5%. But more refined estimates yield much lower fatality rates. After adjusting for lags in the reporting of deaths and differences in fatality rates by age, China’s fatality rate from COVID-19 has been estimated to be in the range of 1.1% (Russell et al., 2020) to 1.4% (Verity et al., 2020; Guan et al., 2020). Further adjustments to include estimates of asymptomatic cases in the denominator yield even lower fatality rates—in the range of 0.5%–0.7%.

### 6.2 Italy

The bottom panels of Fig. 5 show the out-of-sample predictions for Italy. At the end of our data sample, there are about 12,400 infected cases and about 232,000 closed cases. The peak number of infections occurred on April 19 (t=54) at 108,165. The projected number of closed cases at the end of the epidemic is around 260,000.

At the end of our data sample, Italy has recorded a total of 35,045 deaths out of 231,994 closed cases, yielding a closed case fatality rate of 15.1%, well above the 5.5% closed case fatality rate for China. Rinaldi and Paradisi (2020) use population level statistics of death records comparing pre-COVID and post-COVID sample periods to estimate a fatality rate of 1.29% for Italy. Using a modified SIR Model, Calafiore, et al. (2020) estimate a fatality rate of 1.18% for Italy using cases that tested positive.

### 6.3 United States

**Fig. 6:**
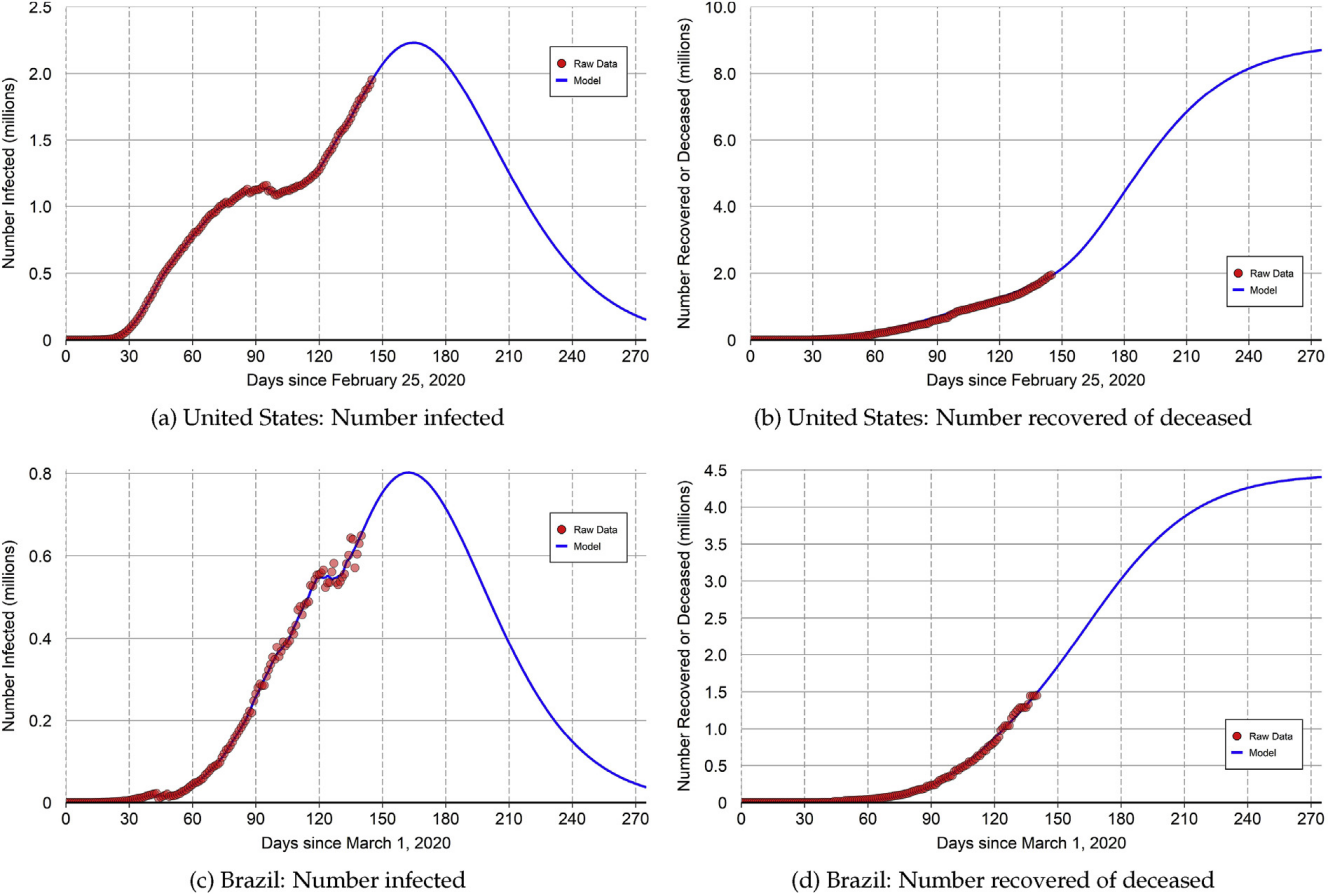
Out-of-sample projections: United States and Brazil. *Notes*: The top panels show the out-of-sample projections for the United States. The peak number of infections is projected to occur on or about August 8 (t=165). The projected number of closed cases at the end of the epidemic is around 8.89 million. The bottom panels show the out-of-sample projections for Brazil. The peak number of infections is projected to occur on or about August 10 (t=162). The projected number of closed cases at the end of the epidemic is around 4.45 million.

The top panels of Fig. 6 show the out-of-sample projections for the United States. At the end of our data sample, there are about 1.953 million infected cases and about 1.946 million closed cases. The number of infected cases reached a local peak on May 30. But after trending down for five days, the number of infections reversed course and has continued to rise through the end of our data sample. The peak number of infections is projected to occur on August 8 (t=165) at about 2.23 million. This projection reflects what might be called a “resurgent first wave” because the plot of the actual and projected number of infections (top left panel of Fig. 6) exhibits a double-peaked shape.

The projected number of closed cases at the end of the epidemic is around 8.89 million (top right panel of Fig. 6). The calibrated value of $\theta_{T}$ for the United States is well below 1.0 and the peak number of infections has yet to be reached. Consequently, the projected number of closed cases at the end of the epidemic is somewhat sensitive to the value of the speed-of-convergence parameter κ that appears in equation (5).^27^ Our baseline projection of 8.89 million closed cases employs κ=0.07. When κ=0.04, the projected number of closed cases declines to around 7.88 million. When κ=0.10, the projected number of closed cases rises to around 9.37 million.

At the end of our data sample, the United States has recorded a total 143,289 deaths out of 1,945,627 closed cases, yielding a closed case fatality rate of 7.4%, somewhat above the 5.5% closed case fatality rate for China. According to the U.S. Centers for Disease Control and Prevention, the best estimate of the overall infection fatality rate for COVID-19 is 0.65%.^28^

On July 20, 2020, the University of Washington’s Institute for Heath Metrics and Evaluation (IHME) was projecting about 225,000 total deaths for the United States for the period through November 1, with an uncertainty range of about 197,000 to 268,000 deaths.^29^ Prior to May 4, 2020, IHME employed a purely phenomenological model that fitted a statistical distribution to the hump-shaped curve of daily deaths in various locations and then used the fitted distribution to project out-of-sample. Starting on May 4, 2020, the IMHE projection methodology was augmented to include a SEIR model component in which the effective reproduction number is allowed to vary over time to closely match the observed number of deaths in each location.^30^ Upon introduction of these updates, the projected number of total deaths from COVID-19 for the United States jumped from 72,433 to 134,475. This example helps to illustrate the wide range of uncertainty surrounding out-of-sample projections, even when constructed by professional epidemiologists.^31^

### 6.4 Brazil

The bottom panels of Fig. 6 show the out-of-sample projections for Brazil. At the end of our data sample, there are about 649,000 infected cases and about 1.45 million closed cases. The peak number of infections is projected to occur on August 10 (t=162) at about 802,000. The projected number of closed cases at the end of the epidemic is around 4.45 million.

At the end of our data sample, Brazil has recorded a total 79,533 deaths out of 1,285,663 closed cases, yielding a closed case fatality rate of 5.5%, the same as China. An epidemiological study of COVID-19 deaths by Ganem et al. (2020) estimates a case fatality rate of 1.6% for Brazil.

### 6.5 Population-adjusted statistics

The four countries we examine have large differences in population, which can affect the total number of cases and the number of resulting deaths from COVID-19. Table 2 provides population-adjusted statistics for the total number of cases (infected plus closed) and the total number of deaths for each country. As before, we use the population of Hubei Province to compute the statistics for China because that area accounts for nearly all confirmed cases. Table 2 shows that China has the lowest number of population-adjusted cases whereas the United States has the highest number. China also has the lowest number of population-adjusted deaths whereas Italy has the highest number.

**Table 2:** Population-adjusted statistics.

	China (H.P.)	Italy	United States	Brazil
Total cases/million	1,394	3,942	11,743	9,905
Total deaths/million	77	565	432	375

*Notes*: Total cases are active cases (currently infected) plus closed cases (recovered or deceased). Statistics are computed using raw data that runs through July 19. H.P. = Hubei Province.

### 6.6 Sensitivity of out-of-sample projections

**Fig. 7:**
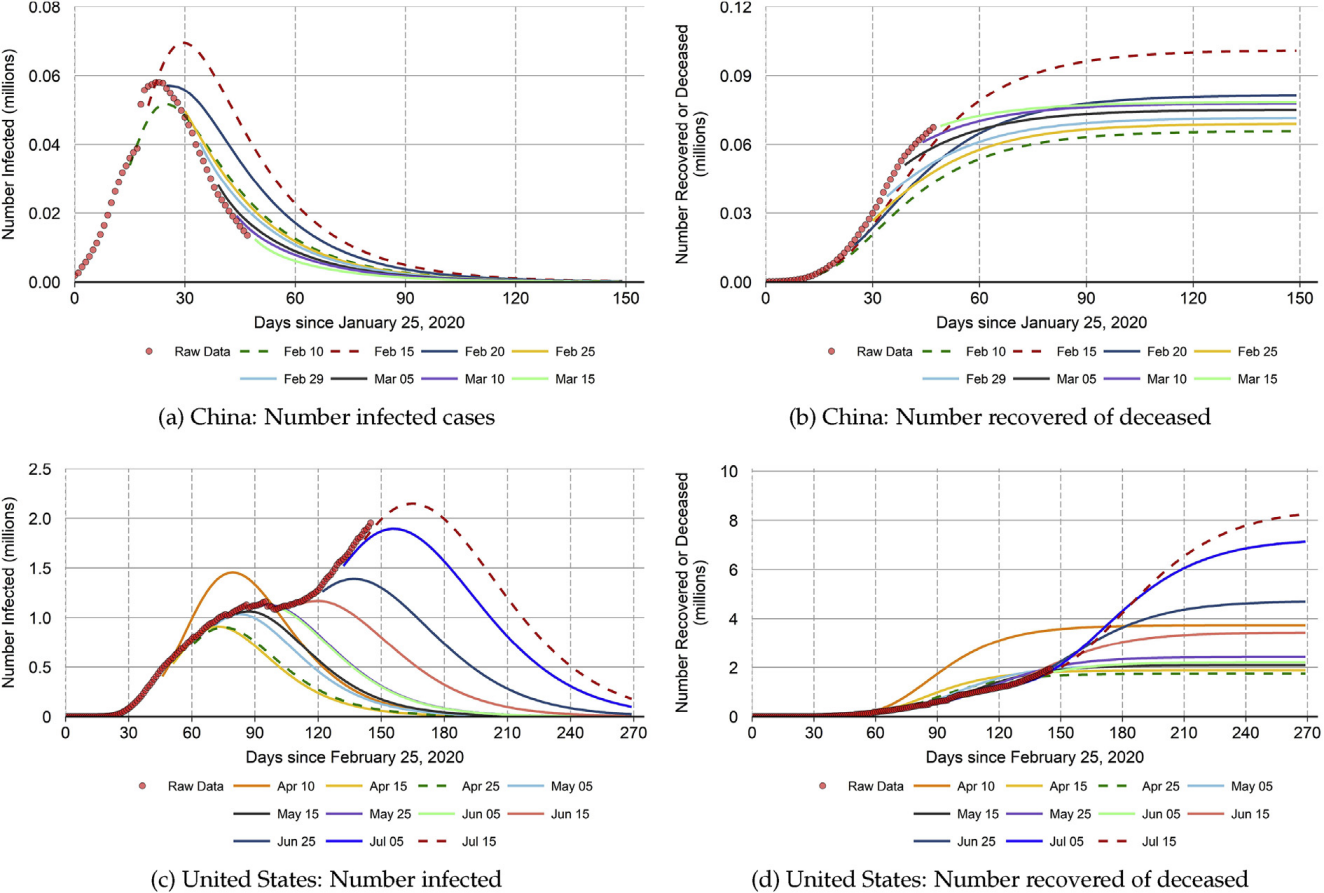
Quasi real-time projections. *Notes*: The figure plots sequences of “quasi real-time” projections for the number of infected cases and the number of closed cases in China and the United States. Each projection uses a different end-of-sample starting point indicated by the month-day label. For each end-of-sample starting point, we recalibrate the values of $\theta_{T},R_{0}$, and η according to the procedures described in Section 4. The out-of-sample projections can sometimes shift by large amounts from one week to the next, depending on recent incoming data. Dashed lines mark the highest and lowest out-of-sample projections for the number of closed cases at the end of the epidemic.

Our out-of-sample projections are subject to enormous uncertainty and can sometimes shift by large amounts from one week to the next, depending on recent incoming data. This is a typical feature of epidemiology (and economic) prediction models.^32^ Fig. 7 illustrates this important point. Specifically, we plot a sequence of “quasi real-time” projections for the number of infected cases and the number of closed cases in China and the United States.^33^ Each projection uses a different end-of-sample starting point. For each end-of-sample starting point, we recalibrate the values of $\theta_{T}$, $R_{0}$, and η according to the procedures described in Section 4.

The left-side panels in Fig. 7 show that our out-of-sample projections can significantly underpredict or overpredict the number infected cases during the early stages of the epidemic when the model-implied $R_{t}$ is above 1.0 and highly volatile. But as the epidemic evolves and the model-implied $R_{t}$ declines and becomes less volatile, the out-of-sample projections exhibit less sensitivity to incoming data. The sensitivity to incoming data also declines after the peak number of infections has been reached. Similarly, Fernandez -Villaverde & Jones, 2020 find that their out-of-sample projections for daily deaths from COVID-19 become less noisy after the peak number of daily deaths in a given location has been reached.

The right-side panels of Fig. 7 show that shifts in the projected trajectory of infected cases can translate into large shifts in the projected number of closed cases at the end of the epidemic (and correspondingly large shifts in the projected number of total deaths). This result highlights the difficulty of formulating a set of health policy containment measures that strike the appropriate balance between epidemiological benefits and the costs that derive from negative impacts to the economy and other health metrics. We note that recent studies of optimal COVID-19 containment policy often treat key model parameters, such as the disease transmission rate, as known constants, thereby suppressing a major source of uncertainty. Hornstein (2020) is an example of one study that does take into account the uncertainty regarding COVID-19 disease parameters. He shows that model-projected outcomes for total deaths as a fraction of the population can vary by a factor of nine.

### 7 Mobility indices and model-implied reproduction numbers

What accounts for the declines in the model-implied reproduction numbers plotted in Fig. 1 through 4? A number of studies have linked declines in daily COVID-19 infections, deaths, or effective reproduction numbers to both mandatory and voluntary containment measures. For example, Xu, et al. (2020) argue that there were two turning points of daily new infections or deaths in the United States which appear to be linked to the implementation of stay-at-home orders in 10 states on March 23 and the Center for Disease Control’s recommendation for the wearing of face-masks on April 3. A study by Pei et al. (2020) of major United States metropolitan areas estimates significant declines in reproduction numbers that appear linked to declines in real-time mobility indices. Maloney and Taskin (2020) present evidence that reductions in mobility for various countries (as measured by Google mobility indices) are driven mainly by voluntary responses. A cross-country study by Deb et al. (2020) finds that daily numbers of infected cases and deaths declined in the 30 days following the implementation of government-mandated containment measures.^34^ Based on trends in Google mobility indices, Hatzius et al. (2020) conclude that voluntary social distancing started in many places before mandatory government controls were enacted, possibly due to fear of the virus.

**Fig. 8:**
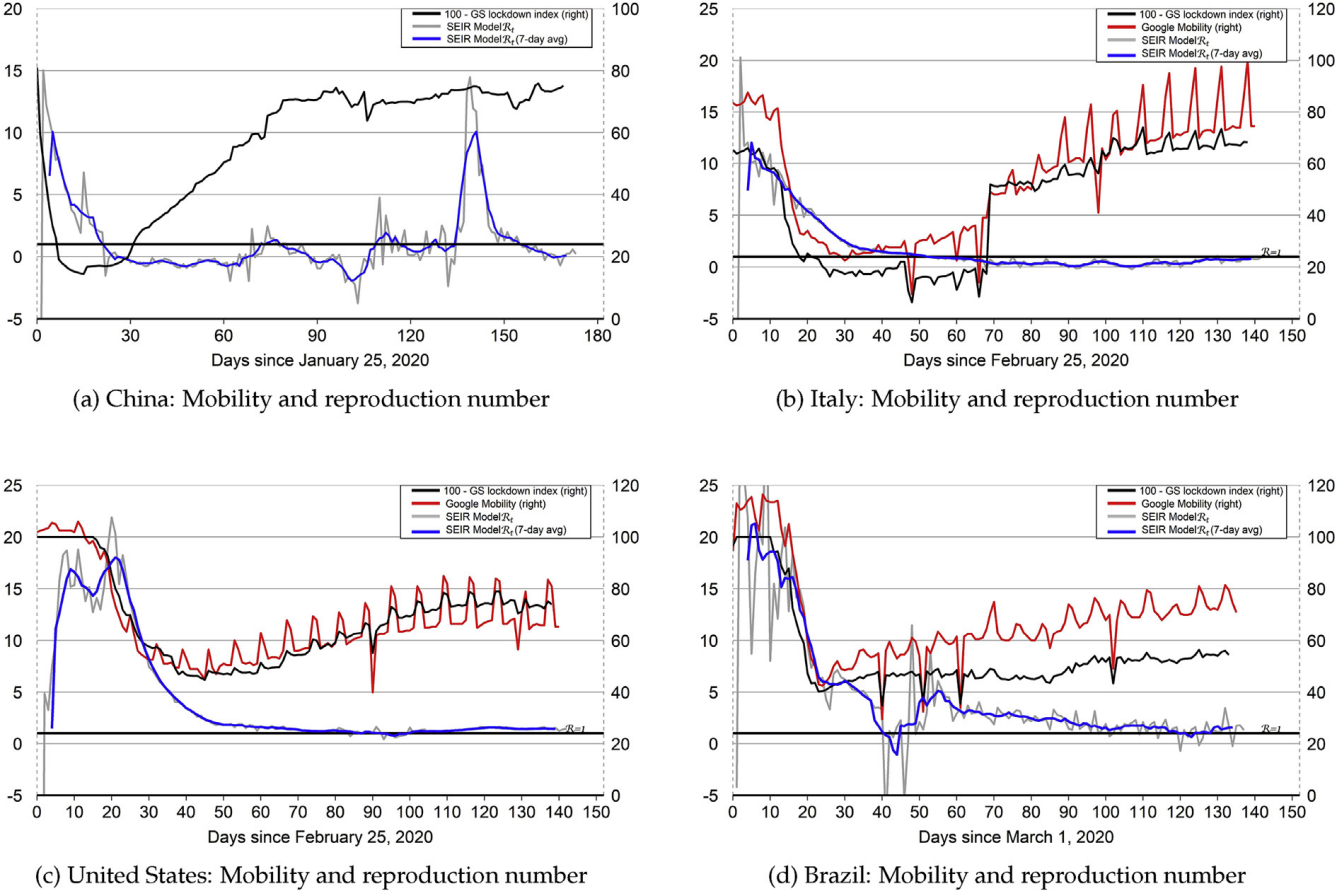
Mobility indices and model-implied reproduction numbers. *Notes*: Declines in measures of population mobility tend to precede declines in the model-implied $R_{t}$ for each country. This pattern suggests that mandatory and voluntary stay-at-home behavior and social distancing during the early stages of the epidemic worked to reduce the effective reproduction number and mitigate the spread of COVID-19. For plotting purposes, the Google mobility indices are re-normalized to have baseline value of 100 instead of zero.

Motivated by the studies mentioned above, Fig. 8 plots the model-implied $R_{t}$ in each country versus measures of population mobility. We use two measures of population mobility: (1) the daily average of the Google mobility indices for workplace and transit locations, and (2) an index defined as 100 minus the Goldman Sachs lockdown index. The Google mobility indices, which do not cover China, are expressed as a percent deviation from a baseline value of zero. For plotting purposes, we re-normalize the baseline value to equal 100.^35^ The Goldman Sachs lockdown index combines lockdown and social distancing measures from the University of Oxford’s Coronavirus Government Response Tracker with Google mobility indices. For China, the lockdown index makes use of subway transportation data.^36^

Fig. 8 shows that declines in measures of population mobility tend to precede declines in the model-implied $R_{t}$ for each country. This pattern suggests that mandatory and voluntary stay-at-home behavior and social distancing during the early stages of the epidemic worked to reduce the effective reproduction number and mitigate the spread of COVID-19.

More recently, measures of population mobility have been trending upwards in all four countries. This pattern reflects both the relaxation of mandatory containment measures and increased voluntary mobility.^37^ As of July 19, a resurgence of new infections in some areas of the United States has triggered a reinstatement of some containment measures, consistent with our behavioral hypothesis set forth in equation (7). At the end of our data sample, measures of population mobility for the United States appear to have plateaued at a level that is below the pre-epidemic baseline.

## 8 Conclusion

Modeling the evolution of COVID-19 is fraught with challenges. There is an enormous range of uncertainty surrounding the projected numbers of infections, recoveries, or deaths. At the same time, this enormous uncertainty highlights the potentially large risks of relaxing containment measures too early. Some countries, including the United States, which had started to relax containment measures are now reversing course after seeing a resurgence in the number of infected cases.

Previous influenza pandemics have typically been followed by a second (and sometimes even a third) wave of infections (Moore et al., 2020). A second wave of infections could be magnified by “seasonal forcing” that serves to push up the effective reproduction number for COVID-19 during the Fall of 2020 (Kissler, Tedijanto, Lipsitch, & Grad, 2020). Some infectious disease experts advocate for maintaining strict containment measures long after the effective reproduction number drops below 1.0.^38^ This is because a delayed relaxation date permits the number of infected cases to be driven much lower, resulting in a slower spread of the disease when random mixing between infected and susceptible groups eventually recommences. Clearly, there are epidemiological benefits of maintaining strict containment measures, but these epidemiological benefits must be balanced against the economic costs and the collateral health damage costs of doing so.

## Appendix Extended model with asymptomatic cases

According to the U.S. Centers for Disease Control and Prevention, the best estimate of the percentage of COVID-19 infections that are asymptomatic is 40%.^39^ Following Aguilar et al. (2020), this appendix extends our model to allow a fraction of infected cases to be asymptomatic. We show that a model that does not explicitly account for asymptomatic cases when they are in fact present can nevertheless capture the impact of asymptomatic cases on the model-implied reproduction number in a reduced-form way. The laws of motion for the generalized model are given by:

(A.1) $$S_{t}=S_{t-1}-{\tilde{R}}_{t}\gamma \frac{S_{t-1}\left(I_{t-1}^{s}+I_{t-1}^{a}\right)}{N},$$

(A.2) $$E_{t}=\left(1-\sigma \right)E_{t-1}+{\tilde{R}}_{t}\gamma \frac{S_{t-1}\left(I_{t-1}^{s}+I_{t-1}^{a}\right)}{N},$$

(A.3) $$I_{t}^{s}=\left(1-\gamma \right)I_{t-1}^{s}+\left(1-\alpha \right)\sigma E_{t},$$

(A.4) $$I_{t}^{a}=\left(1-\gamma \right)I_{t-1}^{a}+\alpha \sigma E_{t},$$

(A.5) $$R_{t}=R_{t-1}+\gamma \left(I_{t-1}^{s}+I_{t-1}^{a}\right),$$

where the superscripts *s* and *a* denote symptomatic and asymptomatic infected cases, respectively. The parameter α is the fraction of exposed cases that are infected without showing any symptoms, i.e. the probability of becoming an asymptomatic case. The effective reproduction number in the generalized model is given by ${\tilde{R}}_{t}\equiv {\tilde{\beta }}_{t}$/γ, where we have assumed that the daily transmission rate and the average illness duration are the same for both types of infected cases.^40^

Solving equations (A.1) through (A.4) for ${\tilde{R}}_{t}$ yields

(A.6) $${\tilde{R}}_{t}=\frac{\sigma^{-1}\left[I_{t}^{s}-\left(1-\gamma \right)I_{t-1}^{s}\right]-\left(1-\sigma \right)E_{t-1}+\sigma^{-1}\left[I_{t}^{a}-\left(1-\gamma \right)I_{t-1}^{a}\right]}{\gamma S_{t-1}\left(I_{t-1}^{s}+I_{t-1}^{a}\right)/N},$$

which collapses to equation (6) when $I_{t}^{a}=I_{t-1}^{a}=0.$ The above expression implies $\partial {\tilde{R}}_{t}/\partial I_{t}^{a}\;>\;0$, i.e., an increase in asymptomatic cases serves to magnify the effective reproduction number for any given values of $I_{t}^{s},$ $I_{t-1}^{s},$ $I_{t-1}^{a}$, and $E_{t-1}$. We can rewrite equation (A.6) as follows

(A.7) $${\tilde{R}}_{t}=\frac{R_{t}\gamma S_{t-1}I_{t-1}^{s}/N+\sigma^{-1}\left[I_{t}^{a}-\left(1-\gamma \right)I_{t-1}^{a}\right]}{\gamma S_{t-1}\left(I_{t-1}^{s}+I_{t-1}^{a}\right)/N},$$

where $R_{t}$ is the model-implied reproduction number from equation (6) in the reduced-form model that does not account for asymptomatic cases. Solving equation (A.7) for $R_{t}$ yields:

(A.8) $$R_{t}={\tilde{R}}_{t}\left(1+I_{t-1}^{a}/I_{t-1}^{s}\right)-\frac{\sigma^{-1}\left[I_{t}^{a}-\left(1-\gamma \right)I_{t-1}^{a}\right]}{\gamma S_{t-1}I_{t-1}^{s}/N}.$$

Equation (A.8) implies that $R_{t}\;>\;{\tilde{R}}_{t}$ whenever ${\tilde{R}}_{t}\;>\;\sigma^{-1}\left[I_{t}^{a}-\left(1-\gamma \right)I_{t-1}^{a}\right]/\left(\gamma S_{t-1}I_{t-1}^{a}/N\right).$ In other words, if the reproduction number ${\tilde{R}}_{t}$ in the true model with asymptomatic cases is sufficiently high to satisfy this condition, then the model-implied reproduction number $R_{t}$ in the reduced-form model that does not account for asymptomatic cases will be even higher. For example, at the start of the epidemic we have $S_{t-1}/N\approx 1$ (because few individuals are infected) and $I_{t}^{a}\approx I_{t-1}^{a}$ (because infections grow very slowly at the start). In this case, we have $R_{t}\;>\;{\tilde{R}}_{t}$ whenever ${\tilde{R}}_{t}\;>\;\sigma^{-1}$.

## Footnotes

1 The virus responsible for the pandemic is officially named SARS Coronavirus-2 or SARS-CoV-2. The resulting disease, which affects the human respiratory system, has been named Coronavirus disease 2019 or COVID-19.

2 See Avery et al. (2020) for an overview of some modeling techniques that have been applied to the COVID-19 epidemic.

3 The labeling of a particular epidemiology model derives from the various health compartments tracked within the population: *S* = Susceptible, *E* = Exposed, *I* = Infected, R= Removed (or Resolved), and D= Deceased. The standard SEIR model does not distinguish between recovery or death, but instead combines these into the single *R* compartment. A SIR model omits the exposed compartment while a SIRD model places recoveries and deaths into separate compartments.

4 But as of July 19, the number of infected cases in China stands at 251, down from 428 on June 30. These numbers reflect a recent outbreak of new cases in the capital city of Beijing (Source: www.nytimes.com/2020/06/15/world/asia/beijing-coronavirus-outbreak.html). Given that nearly all cases up to June 10 were concentrated in Hubei Province, we continue to view the epidemic in that area to be nearly complete.

5 The model-implied $R_{t}$ can be viewed as a reverse-engineered stochastic shock. For examples of this approach in economics, see Gelain, Lansing, & Natvik, 2018 and Lansing (2019).

6 Interactive versions of more complex SEIR models can be found at http://gabgoh.github.io/COVID/index.html and at https://neherlab.org/covid19/. These models require the user to specify the future time path of $R_{t}$.

7 An early version of our paper was presented internally on April 21, 2020, before we had any knowledge of the two papers mentioned above.

8 The basic SIR model was originally developed by Kermack and McKendrick (1927). The discrete-time SEIR model employed here is adapted from Atkeson (2020a).

9 Typical NPIs include early case identification and contact tracing, isolation of infected or contacted cases by voluntary or forced quarantine, travel bans or restrictions, social distancing, stay-at-home orders, school and park closures, mandatory wearing of face masks, and public service campaigns to increase hand washing.

10 Delamater et al. (2019) and Ma (2020) discuss the difficulties involved in measuring or estimating $R_{0}$ or $R_{t}$. Atkeson (2020c) and others define the effective reproduction number as $R_{t}\equiv \left(\beta_{t}/\gamma \right)S_{t}/N$, which multiplies the normalized transmission rate $\beta_{t}/\gamma$ (a time-varying parameter of the disease) by the ratio $S_{t}/N$ (an endogenous variable). If the disease spreads to a significant fraction of the population, then the ratio $S_{t}/N$ will decline over time, causing the effective reproduction number defined in this way to decline mechanically. Our definition $R_{t}\equiv \beta_{t}/\gamma$ seeks to isolate movements in the effective reproduction number that arise solely from changes in the value of $\beta_{t}/\gamma .$

11 Fine et al. (2011) examine the concept of “herd immunity” from theoretical and practical perspectives.

12 Our discrete-time model approximates the continuous-time derivative for any variable $X_{t}$ as $dX_{t}/dt\approx X_{t}-X_{t-1}.$ In the continuous-time limit, there is no distinction between the value of right-side variables dated either *t* or t−1.

13 An extreme example of this phenomenon can be found in the COVID-19 data for Norway. The reported number of recovered cases remained constant at 32 from mid-April through May 21. On May 22, the reported number of recovered cases jumped to 7,727.

14 The adding-up constraint $S_{t}+E_{t}+I_{t}+R_{t}=N$ is relaxed when $\theta_{t}\neq 1.$ In our model projections, the resulting percentage deviation, defined as $100\times \left(S_{t}+E_{t}+I_{t}+R_{t}-N\right)/N,$ never exceeds 1.6% in absolute value for any country. This deviation can be interpreted as reflecting changes in *N* over time (due to births, deaths, or migration) or errors in measuring $I_{t}$ or $R_{t}$.

15 The model-implied $R_{t}$ can even turn briefly negative if $I_{t}-\left(1-\gamma \right)I_{t-1}<\sigma \left(1-\sigma \right)E_{t-1}.$

16 The data for China shows only 66 infected cases on April 17. But the data for April 16 and April 18 show 1081 and 1058 infected cases, respectively. We interpreted the April 17 number to be a data entry error and recoded it as 1066 infected cases.

17 Regarding data on recoveries, Worldometer states “This statistic is highly imperfect, because reporting can be missing, incomplete, incorrect, based on different definitions, or dated (or a combination of all of these) for many governments, both at the local and national level … In view of this, “Active Cases” and “Closed Cases Outcome” which both depend on the number of recoveries (in addition to an accurate death count and a satisfactory rate of case detection, both of which are lacking in the vast majority of countries) can be affected by this inherent flaw for many countries and for the total worldwide count.” Source: https://www.worldometers.info/coronavirus/about/.

18 According to the Wall Street Journal “A growing pool of global death statistics indicates that few countries are accurately capturing fatalities from the new coronavirus—and in some the shortfall is significant.” Source: https://www.wsj.com/articles/most-countries-fail-to-capture-extent-of-covid-19-deaths-11590658200.

19 Starting on June 25, 2020, the COVID-19 model developed by the University of Washington’s Institute for Heath Metrics and Evaluation (IHME) employs a behavioral function in which the trend of easing containment measures in a given location continues along its current trajectory until the daily death rate rises above a threshold, thus triggering a reintroduction of stricter containment measures. For details, see http://www.healthdata.org/covid/updates.

20 In an earlier version of this paper, we assumed that the out-of-sample reproduction number evolved according to the exogenous law of motion: $R_{t}=R_{0}\text{exp}\left(-\eta t\right)+\left[1-\text{exp}\left(-\eta t\right)\right]R_{\infty },$ with $R_{0}$ and η estimated from in-sample data and $R_{\infty }=0.1$.

21 For the quasi real-time projections plotted in Fig. 7 for the earlier stages of the epidemic cycle in China, the end-of-sample target value is the model-implied $R_{t}$ from equation (6) averaged over the most recent 7 days.

22 Studies that estimate the effective reproductive number in China include Kucharski et al. (2020), Wang et al. (2020), and Wu, Leung, & Leung, 2020.

23 Cerada et al. (2020) provide estimates of the effective reproduction number in regions of northern Italy in February 2020.

24 The raw number of infected cases dropped from 21,929 on April 13 to only 9,704 on April 14. Four days later on April 18, the raw number of infected cases was back up to 20,335. The raw number then dropped to 14,062 on April 19.

25 The first cases were identified in early December 2019. On December 31, 2019, the Wuhan Health Commission notified the China Center for Disease Control and Prevention and the World Health Organization (WHO) of a potential virus problem. On January 23, 2020, travel from Wuhan City was shut down, followed by similar travel shutdowns for 16 other cities in Hubei Province. Sources: Wu and McGoogan (2020), Wang et al. (2020) and Leung et al. (2020).

26 According to Lai et al. (2020): “In Wuhan, where the largest number of infected people live, residents were required to measure and report their temperature daily to confirm their onset, and those with mild and asymptomatic infections were also quarantined in ‘Fang Cang’ hospitals, which are public spaces such as stadiums and conference centers that have been repurposed for medical care.” The early detection and isolation of cases was estimated to prevent more infections than travel restrictions and contact reductions.

27 For the other three countries, the sensitivity of the out-of sample projections to the value of κ is much lower because $\theta_{T}$ is already close to 1.0 (China and Brazil) or because the number of infections is well past the peak (Italy).

28 Source: www.cdc.gov/coronavirus/2019-ncov/hcp/planning-scenarios.html.

29 Daily updates of the projections can be found at https://covid19.healthdata.org/projections.

30 Details of the May 4 update can be found at http://www.healthdata.org/sites/default/files/files/Projects/COVID/Estimation_update_050420.pdf.

31 Atkeson (2020c) provides a simplified example of IHME’s pre-May 4 forecasting approach. He shows that when mapped into the daily number of deaths predicted by a simple SIRD model, the IHME’s approach implies an effective reproduction number that falls linearly over time, possibly resulting in an optimistic forecast if the declining time trend does not materialize in practice. Similarly, Wang et al. (2012) demonstrate a one-to-one mapping between the parameters of a curve-fitting approach based on the Richards (1959) model and a simple SIR model.

32 For epidemiology models, see the record of real-time forecasts from the University of Washington’s Institute of Heath Metrics and Evaluation (IHME) model, which are available from https://www.covid-projections.com.

33 Orphanides and van Norden (2002) employ this quasi real-time methodology to demonstrate that most of the variation in real-time estimates of the output gap (defined as the percent deviation of actual GDP from trend GDP) is due to new incoming data, as opposed to revisions to older data. The COVID-19 data from www.worldometers.info/coronavirus/are frequently revised without any notifications to the user. Taking into account these real-time data revisions would increase the uncertainty surrounding our out-of-sample projections.

34 Data on the various containment measures are from the University of Oxford’s Coronavirus Government Response Tracker: www.bsg.ox.ac.uk/research/research-projects/coronavirus-government-response-tracker.

35 The Google mobility indices are available from https://www.google.com/covid19/mobility/.

36 Data on the Goldman Sachs lockdown index are available from https://research.gs.com/content/research/en/reports/2020/07/15/38f54e72-93ba-4fdd-a166-5781558b43fd.pdf. See also Tilton and Struyven (2020).

37 Chakrabarti and Pinkovskiy (2020) find that the relaxation of mandatory containment measures contributes to increases in mobility after accounting for trends that were already in place at the time of relaxation.

38 See, for example, McBryde et al. (2020) and the following Washington Post news article from April 8, 2020: https://www.washingtonpost.com/national/health-science/as-social-distancing-shows-signs-of-working-whats-next-crush-the-curve-experts-say/2020/04/08/3c720e06-7923-11ea-b6ff-597f170df8f8_story.html.
